# Mechanisms of Cell Non-Autonomous Longevity Regulation

**DOI:** 10.1093/geroni/igab046.2560

**Published:** 2021-12-17

**Authors:** Scott Leiser, Hyo Choi, Hillary Miller, Ajay Bhat, Marshall Howington, Elizabeth Dean, Shijiao Huang

**Affiliations:** 1 University of Michigan, Ann Arbor, Michigan, United States; 2 University of Michigan, University of Michigan, Michigan, United States

## Abstract

An organism’s ability to respond to stress is crucial for long-term survival. These stress responses are coordinated by distinct but overlapping pathways, many of which also regulate longevity across taxa. Our previous work identified a cell non-autonomous signaling pathway led by the hypoxia-inducible factor and resulting in induction of flavin-containing monooxygenase-2 (fmo-2) to promote health and longevity. Our current work identifies a distinct cell non-autonomous pathway downstream of dietary restriction (DR) that also relies on fmo-2 induction to promote health and longevity. We now find that these cell non-autonomous pathways can be mimicked by small molecule interventions that increase longevity by inducing fmo-2. Based on the commonalities of these pathways, we hypothesized that fmo-2, a classically annotated xenobiotic enzyme, might play a key endogenous role in responding to metabolic stress. Our resulting data, using metabolic profiling and further epistatic analysis, both support this hypothesis and link fmo-2’s mechanism to modifications in one-carbon metabolism (OCM), a key intermediate pathway consisting of the folate and methionine cycles. Using mathematical modeling and a labeled metabolomics approach, we were able to further identify the likely mechanism of fmo-2-mediated metabolic effects and connect them to both OCM and downstream components. We propose that fmo-2 is induced cell non-autonomously to modify systemic metabolism and longevity, and that fmo-2 is a key member of a conserved metabolic stress response.

